# Assessment of Lead (Pb) Remediation Potential of Senna obtusifolia in Dareta Village, Zamfara, Nigeria

**DOI:** 10.5696/2156-9614-10.25.200301

**Published:** 2020-01-22

**Authors:** Udiba Ugumanim Udiba, Ekpo Eyo Antai, Ekom Robert Akpan

**Affiliations:** 1 Department of Zoology and Environmental Biology, University of Calabar, Calabar, Nigeria; 2 Institute of Oceanography, University of Calabar, Calabar, Nigeria

**Keywords:** remediation potential, *Senna obtusifolia*, lead, Dareta Village, translocation factor, bioaccumulation factor

## Abstract

**Background.:**

Environmental contamination by lead (Pb) and other toxic metals is of significant environmental and human health concern. Heavy metals are not readily eliminated by degradation, and thus remediation of contaminated media (soil, sediment and water/sludge) requires the outright removal or cleanup of these metals. Evaluation of the performance and cost efficiency of various remediation methods has led to the development of bioremediation as an inexpensive, innovative and environmentally friendly cleanup strategy.

**Objectives.:**

The present study was designed to assess the Pb remediation potential of wild Senna obtusifolia (Sicklepod), in Dareta Village, Zamfara, Nigeria.

**Methods.:**

Soil and Senna obtusifolia samples were collected from established plots and Pb content was determined using a Shimadzu atomic absorption spectrophotometer (model AA-6800, Japan) after wet digestion.

**Results.:**

The mean concentrations of Pb (mg/kg) in soil, roots, stems and leaves, respectively, were 130.68±5.2, 61.33±17.86, 66.64±18.10 and 173.39±13.73 for plot 1, 287.84±6.5, 69.42±11.62, 123.4±3.67 and 294.28±4.38 for plot 2, 315.73±4.13, 68.42±10.22, 86.89±6.08 and 290.61±7.47 for plot 3, 396.86±5.48, 91.64±2.87, 150.58±2.21 and 282.53±5.69 for plot 4 and 264.23±8.02, 72.71±2.18, 124.60±2.27 and 282.40±3.79 for plot 5. Average values for the translocation factor, bioaccumulation factor and bioconcentration factor were 3.65±0.66, 1.01±0.23 and 0.29±0.10, respectively.

**Discussion.:**

Soil Pb levels in the present study were found to be within the United States Environmental Protection Agency (USEPA) standards and the Dutch Intervention Values for Pb in soil. Lead content of Senna obtusifolia leaves was found to be higher than the Pb content of the stem and root, indicating relatively low restriction and the efficiency of internal transport of the toxic metal from the roots towards the aerial parts. High translocation and bioaccumulation factors indicate that the plant has vital characteristics for phytoextraction of Pb. The mean Pb concentration of Senna obtusifolia leaves was found to be far above Codex general standards and the European Union (EU) maximum levels for Pb in leafy vegetables.

**Conclusions.:**

The study concludes that wild Senna obtusifolia has significant characteristics for phytoextraction of Pb and that consumption of Senna obtusifolia leaves from the study area would pose a serious risk of Pb intoxication.

**Competing Interests.:**

The authors declare no competing financial interests.

## Introduction

Environmental contamination by heavy metals such as lead (Pb) or cadmium (Cd) is of significant concern. These metals cannot be destroyed by degradation, and thus remediation of contaminated medium requires outright removal or cleanup.^1^ A number of technologies are available to clean up metal-contaminated environments. However, a majority of these technologies are costly to implement and may cause further disturbance to the already damaged environment. Evaluation of the performance, cost implication and public acceptability of various methods applied to clean up different types of pollutants from the environment has led to the development of bioremediation as an evolving, cost-effective, innovative and environmentally friendly cleanup strategy. Bioremediation entails the use of living organisms for the recovery or cleanup of a contaminated medium (soil, sediment, sludge or water) and is defined by Phillips as any process that uses living organisms (microorganisms, fungi, green plant or enzymes) to return a medium altered by a contaminant to its original condition.^2^ Despite this broad definition, bioremediation usually refers specifically to the use of microorganisms for the cleanup of a contaminated medium.^3^ The use of green plants to clean up contaminated sites is referred to as phytoremediation and is considered a form of bioremediation. The process of phytoremediation is an emerging green technology for the cleanup of toxic chemicals in soil, sediment, ground water, surface water, and air.^4^ The use of metal-accumulating plants to remove heavy metals and other compounds was first introduced in 1983, but the concept has been implemented for the past 300 years on wastewater discharges.^3^,^1^ The technique relies heavily on the use of plant interactions in the contaminated site to mitigate the toxic effects of pollutants.^5^ The complex chemical, physical and biological interactions that take place within the medium adjacent to plant roots allow for cleanup of contaminated sites through a number of phytoremediation mechanisms.^4^,^6^ The phytoremediation mechanism most commonly employed for the treatment of heavy metal-contaminated soil involves the use of green plants to absorb, concentrate and precipitate contaminants from the soil into the above ground parts of the plant (phytoextraction). One of the major advantages of this remediation approach is that some precious metals can bioaccumulate in plants and be recovered after remediation, a process known as phytomining.^5^ With ever increasing global metal contamination, plant remediation provides efficient, cost effective and ecologically sound approaches for sequestration and removal through leaves, stem and roots.^7^ The success of the phytoextraction process, whereby pollutants are effectively removed from soil, is dependent on an adequate yield of plants and/or the efficient transfer of contaminants from the roots of the plants into their aerial parts.^8^,^9^ The discovery of plant species capable of accumulating 100 times more metals (hyperaccumulators) than other non-accumulating plants in the same medium demonstrates that plants have significant potential to remove metals from contaminated soil and thus bioremediation is considered a green alternative to the problem of heavy metal pollution.

**Abbreviations**
*ANOVA*: Analysis of variance
*BAC*: bioaccumulation factor
*BCF*: Biological concentration factor
*TF*: Translocation factor

Plants with exceptional metal accumulating capacity (hyperaccumulators) occur throughout the plant kingdom.^9^ Globally, the discovery of hyperaccumulating plants has been slow-footed due to a lack of systematic screening of plant species in several regions of the world.^10^ In 2017, a global data base for plants that hyperaccumulate metal and metalloids shows that about 721 hyperaccumulator species representing over 45 families have been documented.^10^ Currently, they are only a few known hyperaccumulators for Pb; eight plant species belonging to six families.^10^ Melastoma melabathricum for instance has been shown to accumulate up to 13 800 mg/kg in the root of the plant, but only a small amount of the Pb could be translocated from the root to the above ground part (translocation factor (TF) <1), indicating that it is a good bioaccumulator (bioavailability factor >1) of the toxic metal and that phytostabilization is the mechanism at work in the uptake of Pb.^11^ However, certain species of the Noccaea genus and other plants have been identified to have the potential to uptake Pb and transport it from the roots to the shoot. Noccaea praecox has been reported to accumulate over 4000 mg/kg Pb in the shoot, while Noccaea rotundifolia and Noccaea caerulescens have recorded Pb uptake greater than 28 700 mg/kg and 65 631 mg/kg, respectively.^9^

Once introduced into the soil, Pb is difficult to remove. The heavy metal is strongly bounded to soil and all interactions within the soil matrix are pH dependent. Under acidic conditions (pH < 5.5) Pb is more mobile and readily available to plants. Lead is one of the constituents that makes up the earth's crust in nature. The metal is commonly found in water, soil and plants at barely detectable levels.^12^ In nature, the occurrence of metallic Pb is rare. The principal ores of Pb are cerussite (PbC0_3_) and galena (PbS). Other ore minerals such as pyromorphite (Pb_5_(PO_4_)_3_Cl) and anglesite (PbSO_4_) also occur frequently, but are less important.^12^ Lead is often present as a constituent of ores that contain gold, silver, as well as copper, and is usually obtained “as a co-product of these metals”.^13^ Due to anthropogenic activities, Pb has come to be known as the most widely scattered toxic metal in the world.^14^ The accumulation of Pb in plants depends upon the species, plant cultivar, plant organ, the exogenous concentration of Pb and the presence of other ions in the environment. The accumulated Pb content generally increases with the increase in the metal in the environment.^15^

Mass acute Pb pollution and poisoning was reported in Zamfara state in 2010. The source of the widespread poisoning was traced to artisanal gold mining and ore processing in the villages. Lead concentration exceeding 100 000 mg/kg, far above 400 mg/kg considered acceptable for residential areas, was reported in Dareta common areas including residential compounds. Over 735 children were reported to have died and thousands sickened by the toxic metal in what is believed to be the worst Pb poisoning worldwide in the last forty years.^16^ Mineral ore processing activities involving crushing, washing and gold recovery were carried out at many sites within the residential areas in the affected villages. An immediate medical response protocol was developed to provide oral chelation therapy to children between the ages of 0–5 years, pregnant women and breast-feeding mothers. In order not to compromise the efficacy of the chelation therapy, immediate remediation of the affected villages was carried out.^17^ The remediation of Dareta village (perhaps the most affected village) took place from June to July 2010. The remediation was a simple process involving the removal of 5 cm of contaminated topsoil in areas with soil Pb level greater than 1000 mg/kg and replacing with uncontaminated soil. The excavated contaminated topsoil was then buried in landfills. Areas with soil Pb levels between 400–1000 mg/kg were simply covered with about 8 cm of clean soil and compacted. Immediately after the remediation exercise, soil Pb levels were reported in the village in the range of 81.65–684.27 mg/kg, indicating over 95% reduction in soil Pb levels.^18^,^19^ Three years after the remediation exercise, Udiba *et al.* recorded soil Pb levels ranging from 1029.42±98.50 mg/kg to 6724.68±184.00 mg/kg in the area and observed that the old grinding mill and other areas where ore processing took place were eventually covered with wild Senna obtusifolia growing aggressively and stifling the growth of preexisting plants.^20^

The present study was designed to assess the Pb remediation potentials of wild Senna obtusifolia, in Dareta Village, Zamfara State, Nigeria. Despite the high soil Pb concentrations recorded, areas covered with wild Senna obtusifolia were found to be within permissible limits, hence the need to investigate the phytoremediation potentials of the plant. A fast growth rate, high above ground biomass, tolerance to Pb pollution, survival and adaptability to prevailing environmental conditions exhibited by wild Senna obtusifolia in Dareta village are some of the important characteristics to consider when choosing a plant to phytoremediate Pb in soil.^21^,^22^ The ability of this plant to cleanup Pb-contaminated sites depends on the amount of metals that can be accumulated by it, soil Pb concentration, rate of uptake, and translocation and accumulation in harvestable tissues. These are important properties for phytoextraction of toxic metals, which this study was designed to investigate. The success of any phytoremediation approach is anchored primarily on optimizing the remediation potentials of native plants growing in polluted sites. The findings of the present study may present the need for optimizing the remediation potentials of Senna obtusifolia. Putshaka *et al*. has previously reported Senna obtusifolia to accumulate 81.50 mg/kg Pb, a value that is greater than the corresponding soil Pb concentration in the flood plain of a tannery wastewater stream in Challawa industrial estate, Kano.^23^

## Methods

**Figure 1:**
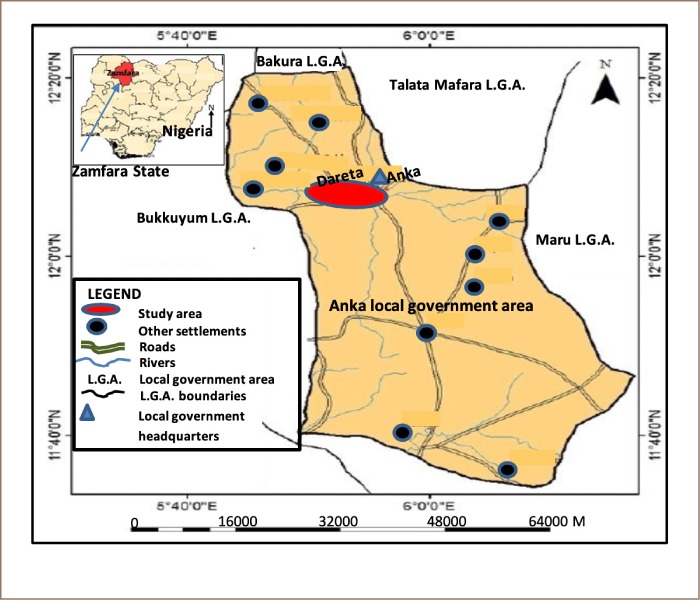
Map of Anka Local Government Area of Zamfara State, Nigeria, showing Dareta Village with sampling points

Zamfara State is located in the northwest geopolitical zone of Nigeria. It occupies a land mass of about 39 762 sq km and Gusau is its capital. The state shares boundaries with Sokoto, Niger, Kebbi, Katsina and Kaduna State within the country and an international boundary with Niger Republic *(Figure 1)*.^24^

The climate of this northwestern Nigerian State is warm and tropical, with temperatures sometimes rising up to 38°C at the peak of the dry season (between March and April). The rains begin between mid-March and May, lasting for about six months up until end of October, while the extremely dry, cold and dusty wind that blows from the Sahara towards the western coast of Africa (Harmattan) lasts from December to February. The mean annual rainfall in the area fluctuates between 36 and 80 mm, varying slightly, from the northern to the southern parts of the state. The vegetation of the state is made up of Sudan and Guinea Savannah. The Sudan Savannah is found in the northern, western and eastern part of the state. The Northern Guinea Savannah is found in the southern part of the state. This vegetation type is largely found in the Gusau, Tsafe and Anka Local Government Areas.^25^

Dareta village (the study area) is in the Anka Local Government Area, located on 12°06′30′ N 5°56′00′ E with total area of about 2746 sq km and a human population of about 142 280.^26^ Based on the 2006 Anka Local Government Area census data, the estimated population of Dareta village is 1033 and number of children less than 5 years old, based on 20% of population, is 207. The village is primarily populated by the Hausa and Fulani people. Until recently, following the discovery of gold mines, the main activities of the people of Dareta village was farming. Recently artisanal mining activities has engaged a large percentage of the population.

### Sample collection and preservation and preparation

Five different plots were carefully selected for the study and designated as plots 1, 2, 3, 4 and 5. Three sampling points were established in each plot. The plants were uprooted carefully and soil samples collected from the same points. Soil samples were collected at the surface level (0–10 cm depth) in duplicates using a soil auger. Additionally, some Senna obtusifolia was harvested at the control site in Zaria, Kaduna State and soil samples collected from the same points. Samples were preserved in black polyethene bags and transported to the Environmental Laboratory, National Research Institute for Chemical Technology, Zaria, Nigeria. Leaves, stems and roots of Senna obtusifolia were thoroughly washed to remove all adhered soil, cut into pieces and air dried for 5 days in the laboratory. The dried leaves samples were pulverized, and stems and roots were pounded before sieving through 1 mm mesh and digested. The digestion of 1g was carried out using 5 ml of concentrated nitric acid according to Awofolu.^27^

Soil samples from each plot were thoroughly mixed to obtain a representative sample, air dried, crushed and sieved with 2 mm mesh before wet digestion. One (1) g of a well mixed sample from each sampling point was taken into a 250 ml glass beaker and digested with 10 ml of concentrated nitric acid, perchloric acid and hydrofluoric acid in the ratio 3:1:1 on a hot plate. After evaporating to near dryness, 10 ml of 2% nitric acid was added, filtered into a 50 ml-volumetric flask and then made up to mark with distilled deionized water.^18^

### Sample analysis

Lead concentrations in the digests were determined using a Shimadzu atomic absorption spectrophotometer (model AA-6800, Japan) equipped with Zeaman background correction and graphite furnace at the National Research Institute for Chemical Technology, Zaria, Nigeria. The instrument was then set to 0 by running the respective reagent blanks and Pb concentration was determined at a wavelength of 283.3 nm. An average value of three replicates was taken for each determination. Data obtained were subjected to statistical analysis.

### Analytical quality assurance

Appropriate quality assurance procedures and precautions were taken to ensure the authenticity of the results. Samples were carefully handled to avoid cross-contamination. Glassware was properly cleaned and deionized water was used throughout the study. All of the reagents, including nitric acid (Riedel-de Haen, Germany), hydrofluoric acid (Sigma-Aldrich, Germany) and perchloric acid (British Drug House Chemicals Limited, England) were of analytical grade. In order to check the reliability of the analytical method employed for Pb determination, one blank and combine standards were run with every batch of 12 samples to detect background contamination and monitor consistency between batches. The result of the analysis was validated by digesting and analyzing standard reference materials (Lichen coded IAEA-336) following the same procedure. The analyzed values and the certified reference values of the elements determined were compared to determine the reliability of the analytical method employed.

### Statistical analysis

Test for normality was carried out using the Shapiro-Wilks test, and the Z-score test was used to check for outliers.

### Analysis of variance test

Having passed the test for normality and outliers, data collected were subjected to statistical test of significance using the analysis of variance (ANOVA) test to assess significant variation in Pb concentration in the soil, Senna obtusifolia roots, stem and leaves across the five different plots under study. Probabilities less than 5% (p < 0.05) were considered to be statistically significant. Independent t-test was used to compare Pb content of Senna obtusifolia from the study area and the Pb content of Senna obtusifolia from Zaria (control). Probabilities less than 5% (p < 0.05) were considered to be statistically significant.

### Pearson product-moment correlation coefficient

Pearson product-moment correlation coefficient was used to determine the association between Pb levels in the soil, stem, roots and leaves of Senna obtusifolia at α = 0.05.

All the above-mentioned statistical analyses were performed by SPSS software 17.00 for Windows.

### Bioavailability factor

The bioavailability factor of heavy metals in plants, also known as the bioavailability index, was calculated according to Malik *et al.,* and expressed in Equation 1.^28^

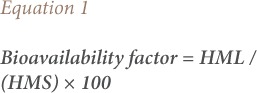
where, HM_L_ is mg of heavy metal per kg of plant leaves and HM_S_ is total content of heavy metal per kg of soil.

### Biological concentration factor, translocation factor and bioaccumulation factor

The biological concentration factor (BCF) was calculated as a metal concentration ratio of plant roots to soil, and is given in Equation 2.^28^

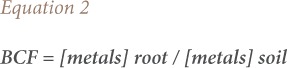



The TF was described as the ratio of heavy metals in plant shoots to that in the roots, as given in Equation 3.^28^

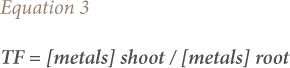



The bioaccumulation factor (BAC) was calculated as the ratio of a heavy metal in plant shoots to that in soil, as shown in Equation 4. 28

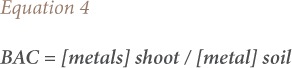



## Results

To evaluate the accuracy and precision of the employed analytical procedure, standard reference materials of Lichen coded IAEA-336 was analyzed in a like manner to the samples in the present analysis. The analyzed values were found to be within the ranges of the certified reference values for the elements in the present study, suggesting the reliability of the employed methods *(Table 1).*

**Table 1:** Analyzed Reference Material Results (Lichen IAEA - 336) Compared to Certified Reference Values (mg/kg)

	**Elements (mg/kg)**				

	**Pb**	**Cd**	**Copper**	**Manganese**	**Zinc**
Analyzed value	5.25	0.140	4.00	55.78	29.18
Reference value	4.2–5.5	0.1–2.34	3.1–4.1	56–70	27–33.80

**Figure 2:**
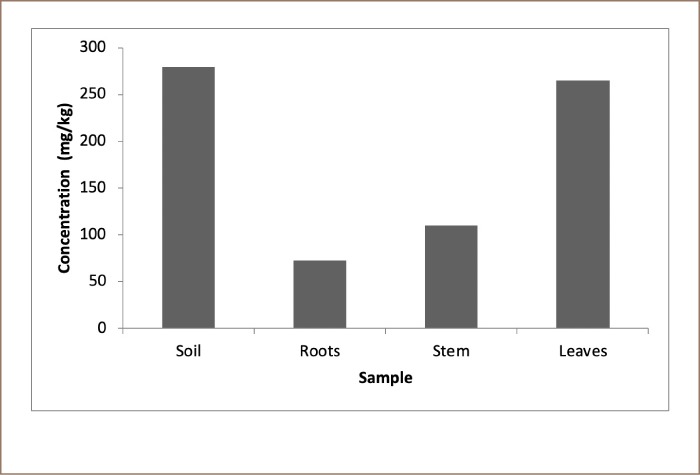
Average Pb levels of soil and Senna obtusifolia tissues, Dareta village

Lead content of soil and Senna obtusifolia tissues across sampling plots in Dareta village are presented in Table 2. Correlations between Pb concentration in soil and Senna obtusifolia tissues are presented in Table 3. Translocation factor, BCF and BAC are presented in Table 4. Average Pb levels of soil and Senna obtusifolia tissues are shown in Figure 2.

**Table 2:** Lead Levels of Soil and Senna obtusifolia Tissues in Dareta Village (mg/kg)

**Senna Obtusifolia plot**	**Sampling point**	**Soil**	**Roots**	**Stem**	**Leaves**
1	1	130.75	52.19	56.85	163.81
	2	135.87	81.91	87.54	189.12
	3	125.41	49.88	55.54	167.23
	Mean ±SD	130.68±5.2^a^	61.33±17.86^a^	66.64±18.10^a^	173.39±13.73^a^
2	1	287.60	71.52	127.095	289.38
	2	281.42	56.89	119.76	295.65
	3	294.49	79.86	123.42	297.82
	Mean ±SD	287.84±6.5^b^	69.42±11.62^a^	123.4±3.67^b^	294.28±4.38^b^
3	1	339.28	72.65	93.80	296.02
	2	346.73	56.76	84.51	282.09
	3	331.19	75.84	82.36	293.72
	Mean ±SD	315.73±4.13^c^	68.42±10.22^a^	86.89±6.08^a^	290.6 l±7.47^b^
4	1	396.64	94.56	152.42	288.23
	2	391.48	88.82	151.19	276.85
	3	402.45	91.53	148.12	282.51
	Mean ±SD	396.86±5.48^d^	91.64±2.87^a^	150.58±2.21^c^	282.53±5.69^b^
5	1	268.37	74.97	126.86	280.43
	2	254.98	72.54	124.62	279.99
	3	269.33	70.63	122.32	286.78
	Mean ±SD	264.23±8.02^e^	72.71±2.18^a^	124.60±2.27^ad^	282.40±3.79^b^
Zaria (control)	1	63.32	3.10	3.98	4.11
	2	74.76	6.01	6.93	6.96
	3	65.43	2.65	2.54	2.84
	Mean ±SD	67.84±6.09^f^	3.92±1.82^b^	4.48±2.28^e^	4.64±1.7°
Standards					0.30^*^

Mean with the different superscripts along a given column indicates a significant (p < 0.05, ANOVA) difference.

^*^ Commission of the European Communities and Codex Standards.^29^,^30^

**Table 3:** Correlations Between Lead Concentration in Soil and Senna obtusifolia Tissues

		Senna obtusifolia soil	Senna obtusifolia roots	Senna obtusifolia stem	Senna obtusifolia leaves
Senna obtusifolia soil	Pearson	1			
	Correlation				
	Sig. (2-tailed)				
	N	15			
Senna obtusifolia roots	Pearson	.659^**^	1		
	Correlation				
	Sig. (2-tailed)	0.008			
	N	15	15		
Senna obtusifolia stem	Pearson	.776^**^	.760^**^	1	
	Correlation				
	Sig. (2-tailed)	0.001	0.001		
	N	15	15	15	
Senna obtusifolia leaves	Pearson	.829^**^	0.447	.690^**^	1
	Correlation				
	Sig. (2-tailed)	0	0.095	0.004	
	N	15	15	15	15

^**^ Correlation considered significant at the 0.01 level (2-tailed).

**Table 4:** Translocation Factor, Bioconcentration Factor and Bioaccumulation Factor of Lead Across Senna obtusifolia Plots

Senna obtusifolia plot	TF	BAC	BCF
1	2.83	1.33	0.47
2	4.24	1.02	0.24
3	4.23	0.92	0.22
4	3.08	0.71	0.23
5	3.88	1.07	0.28
Mean±SD	3.65±0.66	1.01±0.23	0.29±0.10

Table 2 indicates that the concentration of Pb in soil, Senna obtusifolia roots, Senna obtusifolia stem and Senna obtusifolia leaves in the study area ranged from 125.41–402.45 mg/kg, 49.88–94.56 mg/kg, 55.54–152.42 mg/kg and 163.81–297.82 mg/kg, respectively. The highest soil Pb concentration (402.45 mg/kg) was recorded in plot 4, while the lowest (125.41 mg/kg) was recorded in plot 1. The highest and lowest concentration of Pb in Senna obtusifolia roots and stem were also recorded in plot 4 and 1, respectively *(Table 2).* Senna obtusifolia leaves recorded the highest Pb concentration (297.82 mg/kg) in plot 2 and the lowest Pb concentration (163.81 mg/kg) in plot 1. The mean soil Pb level of soil, Senna obtusifolia roots, stem and leaves from Zaria (control) were 67.84±6.09 mg/kg, 3.92±1.82 mg/kg, 4.48±2.28 mg/kg and 4.64±1.7 mg/kg, respectively.

Figure 2 shows that Pb was detected in Senna obtusifolia plots in the order of: soil > Senna obtusifolia leaves > Senna obtusifolia stem > Senna obtusifolia roots.

Statistical analysis revealed a significant (ANOVA, p < 0.05) difference in overall Pb concentration between the soil, Senna obtusifolia roots, Senna obtusifolia stem and Senna obtusifolia leaves, and Pb concentrations in soil were significantly higher than the Pb concentration in roots and stem. The Pb concentration in Senna obtusifolia leaves was also found to be significantly (p < 0.05) higher than in Senna obtusifolia stem and Senna obtusifolia roots. The Pb concentration in Senna obtusifolia stems was found to be significantly (p < 0.05) higher than in the root. The difference in overall Pb concentration between in soil and Senna obtusifolia leaves was not statistically significant (p > 0.05). The differences in Pb concentrations between soil and Senna obtusifolia tissues across the plots were found to be statistically significant (ANOVA, p < 0.05) (*Table 2*).

Table 3 shows the significant (p < 0.01) positive correlation between the concentration of Pb in soil and Senna obtusifolia leaves (r = 0.829), concentration of Pb in soil and Senna obtusifolia stem (r= 0.776), concentration of Pb in soil and Senna obtusifolia roots (r = 0.659), concentration of Pb in Senna obtusifolia roots and Senna obtusifolia stem (r = 0.760) and the concentration of Pb in Senna obtusifolia stem and Senna obtusifolia leaves (r = 0.690).

Table 4 shows that a TF of 2.83, 4.24, 4.23, 3.08 and 3.88 was obtained for Senna obtusifolia plot 1, plot 2, plot 3, plot 4 and plot 5, respectively. The highest TF obtained for Pb in Senna obtusifolia was found in plot 2, and plot 1 recorded the lowest TF for Pb. The translocation factor followed the trend: Senna obtusifolia plot 2 > plot 3 > plot 5 > plot 4 > plot 1. The BAC of 1.33, 1.02, 0.92, 0.71 and 1.07 was computed for Senna obtusifolia plot 1, plot 2, plot 3, plot 4, and plot 5, respectively. The BCF values were 0.47, 0.24, 0.22. 0.23 and 0.28 for plot 1, plot 2, plot 3, plot 4 and plot 5, respectively. Bioaccumulation factor followed the trend: Senna obtusifolia plot 1 > plot 5 > plot 2 > plot 3 > plot 4, while the BCF followed the trend: Senna obtusifolia plot 1 > plot 5 > plot 2 > plot 4 > plot 3.

## Discussion

Lead concentrations (mg/kg) in soil, Senna obtusifolia roots, stem and leaves *(Table 2)* showed that the soil Pb levels across the five plots were all below the United States Environmental Protection Agency (USEPA) guideline for soil Pb levels (400 mg/kg). Nigeria has no standards for soil Pb levels, but the Department of Petroleum Resources adopts the Dutch standards for the assessment of soil pollution in the country. The Dutch soil remediation policy uses target and intervention values to assess soil contamination. The remediation intervention value, used to indicate the Pb level at which the functional properties of the soil to support human, animal and plant life are seriously threatened or impaired, is 530 mg/kg for Pb.

This value represents the soil Pb concentration above which the soil is said to be seriously contaminated. The target value (85 mg/kg) indicates a soil Pb level below which there is sustainable soil quality. It is the soil Pb level that must be attained to fully recover all the functional properties of the soil for humans, animal and plant life, and is thus the bench mark for environmental quality on the assumption of negligible risk to the ecosystem.^31^ The average soil Pb levels recorded in this study were found to be above the Dutch target values, but below intervention values. This observation is in agreement with the Joint United Nations Environmental Protection/United Nations Office for the Coordination of Humanitarian Affairs (JEU) report that stated that the Pb pollution and poisoning crisis in Dareta village is limited to areas where gold ore processing has taken place and has not spread significantly to other areas such as farms.^24^ Soil Pb levels ranging from 81.65 to 684.27 mg/kg were previously reported for Darata village immediately after the remediation exercise.^18^

The observed high Pb levels in Senna obtusifolia leaves, stem and roots, *(Table 2, Figure 2)* despite the fact that soil Pb levels were within the USEPA acceptable limit and Dutch soil remediation intervention values, may suggest that a good proportion of Pb in Dareta soils is present in the mobile phase, thus readily available for uptake by plants. Plants growing in a Pb-enriched environment are able to accumulate large quantities of the metal in their tissues depending on the percentage bioavailabilty/mobility of the toxic metal. Susceptible plant species that are not resistant to the metal usually die off, allowing the resistant ones to thrive. This could possibly explain the fact that the old grinding mill and other areas where Pb processing had taken place in Dareta were overgrown with Senna obutsifolia at the time of this study. The concentration of Pb in Senna obtusifolia leaves did not follow the trend of soil Pb level across the studied plots *(Table 2).* Senna obtusifolia plot 3 recorded the highest mean Pb level in leaves, while Senna obtusifolia plot 5 recorded the lowest mean concentration. The observation may be attributed to differences in the chemical form of Pb present in soils at the different Senna obtusifolia plots and differences in soil chemistry. It also suggests that the bioavailable percentage of total Pb has a significant effect on Pb uptake. Bano reported that Senna obtusifolia growing by the road side accumulates up to 300 mg/g dry weight of Pb.^15^ Mean Pb contents of 4.350, 7.850, 6.400, 7.700 and 6.000 μg/g were reported for a refuse dump, campus, roadside, forest and garden, respectively, in Cassia occidentalis in Ghana.^32^ Lead content in Senna obtusifolia leaves was found to be higher than the Pb content of the stem and the roots, indicating significant accumulation of the metal in leaves. Leaves may therefore be considered to be the major storage organ for Pb in Senna obtusifolia. The large difference in Pb concentrations between roots and leaves indicate relatively low restriction, hence the efficiency of the internal transport of the toxic metal from the roots towards the aerial parts.^33^,^34^ Translocation factor values lower than or equal to 1.033 suggested that plants grown on highly metal contaminated soils behaved as tolerant plants or excluders. Metal excluders accumulate heavy metals from the surrounding medium into their roots, but exclude their transport and entry into the aerial arts, such plants have low potentials for phytoextraction but may be efficient for phytostabilization purposes.^35^ Where the TF is greater than 1.033, the plant species is a hyperaccumulator of the metal.^33^,^35^,^36^ The high mobility of Pb in Senna obtusifolia from root to leaves is reflected by the high TF *(Table 3).* Transfer factors across the Senna obtusifolia plots were all greater than 2. Plant species with TF values greater than 1 are considered suitable for phytoextraction which generally requires translocation of the heavy metals to easily harvestable parts such as shoots.^30^ High roots to shoots translocation of Pb in this study therefore indicates that Senna obtusifolia is characterized by a widely distributed and highly branched root system and has vital characteristics for phytoextraction of the toxic metal.^37^ A similar finding was recorded by Ghosh and Singh.^38^

The phytoextraction potential of a given plant species is essentially determined not only by its TF, but also by the BAC and BCF.^39^,^40^ Bioaccumulation factor values greater than 1 have also been used successfully to evaluate the potentials of plants for phytoextraction and phytostabiliztion.^41^ The average BAC value for the study was found to be 1.01±0.23, indicating that the plant under study has significant potentials for Pb phytoremediation. In evaluating the phytotoxicity of Pb–zinc tailings to big bluestem (Andropogon gerardii V.) and switchgrass (Panicum virgatum L.), Levy *et al.,* reported normal concentrations of Pb in plants with a range from 0.5 mg/kg to 10 mg/kg and phytotoxic concentration from 30 mg/kg to 300 mg/kg.^42^ Senna obtusifolia in the present study showed Pb concentrations far higher than the normal concentration, but within the phytotoxic levels. The results indicate that Senna obtusifolia is tolerant to the toxic effect of the target metal. Given its fast growth rate, high above ground biomass, tolerance to Pb pollution, survival and adaptability to prevailing environmental conditions, widely distributed and highly branched root system, accumulation of the metal from soil and high translocation from root to shoot exhibited by wild Senna obtusifolia in Dareta village, the plant could be considered a green alternative method for the mitigation of Pb pollution and poisoning in Zamfara.

The significantly higher Pb levels observed in tissues of Senna obtusifolia from Dareta village compared to Senna obtusifolia from Bassawa village, Zaria may be attributed to elevated soil Pb levels due the influence of mining and processing of Pb-rich gold ore by artisans in Dareta village. The significant (p < 0.01) positive correlations observed between concentrations of Pb in soil and Senna obtusifolia leaves (r = 0.829), concentrations of Pb in soil and Senna obtusifolia stem (r= 0.776), and concentrations in soil and Senna obtusifolia roots (r = 0.659) indicate that as Pb concentration in soil increases, Pb contents of the leaves, stem and roots of Senna obtusifolia also increases, suggesting that same source may be responsible for the presence of the toxic metal at the determined concentrations. The significant difference of Pb contents in Senna obtusifolia tissues (roots stem and leaves) may be a reflection the preferred storage organ for the metal in the plant.

Senna obtusifolia is a green leafy vegetable and an important delicacy, commonly called ‘tapassa’ among the Hausa of northern Nigeria. The lead content of the leaves recorded in this study (*Table 2*) was found to be generally higher than the permissible levels by the Food and Agriculture Organization of the United Nations and World Health Organization (FAO/WHO) and the European Union (EU) maximum levels of 0.30 mg/kg for Pb in leafy vegetables.^29^,^43^ Lead concentrations of Senna obtusifolia leaves were also higher than the reported human toxicity levels of 1.00 mg Pb/day.^44^ Consumption of these leaves thus poses significant risk of Pb intoxication. High Pb content in blood affects nearly all organs in the human system and is associated with cardiovascular problems, high blood pressure and developmental impairment affecting sexual maturity and the nervous system.^45^ Children are at greater risk of Pb poisoning because their smaller bodies are in a continuous state of growth and development. Common signs and symptoms in children are loss of appetite, abdominal pain, vomiting, weight loss, constipation, anaemia, kidney failure, irritability and behavioral problems.^46^ In pregnant women, elevated blood Pb levels can lead to miscarriage, preterm birth, low birth weight and problems with childhood development.^47^

## Conclusions

The ability of plants to clean up Pb contaminated sites depends on the amount of the metal that can be accumulated by them, soil Pb concentration, rate of uptake, and translocation and accumulation in harvestable tissues. Soil Pb levels in this study were found to be within the USEPA standards as well as the Dutch intervention values. The lead content of Senna obtusifolia leaves was found to be higher than the Pb content of the stem and root, indicating significant accumulation of the toxic metal in the leaves. Senna obtusifolia leaves were therefore noted as the major storage organ for Pb in the plant. The high mobility of Pb in Senna obtusifolia from root to leaves was reflected by a high TF. The high translocation factor and BAC recorded in this study indicates that Senna obtusifolia has vital characteristics which can be used for phytoextraction of the metal. Significantly higher Pb levels in Senna obtusifolia from Dareta village compared to Senna obtusifolia leaves from Bassawa village, Zaria (control) can be attributed to the influence of mining and processing of Pb-rich gold ore by artisans in Dareta. The range of Pb concentrations of Senna obtusifolia leaves was found to be far above the Codex general standards and the EU maximum levels for Pb in leafy vegetables. The Pb content of Senna obtusifolia leaves was also higher than the reported human toxicity levels of 1.00 mg Pb/day. Consumption of Senna obtusifolia leaves from the study area thus poses serious risk of Pb intoxication. The authors recommend a mass literacy campaign to educate the local population on the inherent dangers of consumption of Senna obtusifolia from the area. Optimizing the remediation potentials of wild Senna obtusifolia growing in Dareta is also recommended.
